# Tracking and risk of abdominal and general obesity in children between 4 and 9 years of age. The Longitudinal Childhood Obesity Study (ELOIN)

**DOI:** 10.1186/s12887-022-03266-6

**Published:** 2022-04-12

**Authors:** Honorato Ortiz-Marrón, Maira Alejandra Ortiz-Pinto, Gloria Cabañas Pujadas, José Galo Martínez Mosquera, Marien Lorente Miñarro, Francisca Menchero Pinos, María Ordobás Gavín, Iñaki Galán

**Affiliations:** 1 Epidemiology Service, Directorate-General of Public Health, Ministry of Health, Community of Madrid, Madrid, Spain; 2 Health Center Los Pintores, Ministry of Health, Community of Madrid, Parla, Spain; 3 Health Center Los Alpes, Ministry of Health, Community of Madrid, Madrid, Spain; 4 Health Center José María Llanos, Ministry of Health, Community of Madrid, Madrid, Spain; 5 National Centre for Epidemiology, Institute of Health Carlos III, Madrid, Spain; 6 Department of Preventive Medicine and Public Health, Faculty of Medicine, Autonomous University of Madrid (IdiPaz), Madrid, Spain

**Keywords:** Abdominal and general obesity, Waist circumference, Children, Tracking, Spain

## Abstract

**Background:**

Studies have shown that overweight and obesity conditions tend to be stable from childhood and adolescence to adulthood. Unfortunately, little is known about the evolution of abdominal obesity during childhood. The aim of this study was to evaluate the temporal variations and risk of general and abdominal obesity between 4, 6, and 9 years of age.

**Methods:**

Measurements of children in the ELOIN study taken at the three follow-ups of 4, 6, and 9 years of age were included (*N* = 1,902). Body mass index and waist circumference were recorded via physical examination. General obesity was determined according to the criteria of the World Health Organization (WHO) and abdominal obesity according to the cut-off points proposed by the International Diabetes Federation (IDF). Prevalence ratios (PRs) were estimated by sex and family affluence using generalized estimating equation models and relative risks (RRs) of obesity were obtained via Poisson regression.

**Results:**

The prevalence of general obesity was 5.1%, 9.1%, and 15.6% at 4, 6, and 9 years, respectively, yielding a PR of 3.05 (95%CI: 2.55–3.60) (9 years old relative to 4 years). The prevalence of abdominal obesity was 6.8%, 8.4%, 14.5% at 4, 6, and 9 years, respectively, and the PR was 2.14 (95%CI: 1.82–2.51) (9 years old relative to 4 years). An inverse correlation was observed between both general and abdominal obesity and socioeconomic status. Among participants with general or abdominal obesity at 4 years of age, 77.3% and 63.6% remained in their obesity classification at 9 years, respectively, and 3.4% and 3.5% presented general or abdominal obesity also at 6 and 9 years of age, respectively. The RRs of general and abdominal obesity at 9 years were 4.61 (95%CI: 2.76–7.72) and 4.14 (95%CI: 2.65–6.48) for children classified with obesity at 4 years of age, increased to 9.36 (95%CI: 7.72–11.35) and 9.56 (95%CI: 7.79–11.74) for children who had obesity at 6 years, and up to 10.27 (95%CI: 8.52–12.37) and 9.88 (95%CI: 8.07–12.11) for children with obesity at both 4 and 6 years, respectively.

**Conclusions:**

General and abdominal obesity begin at an early age and increase over time, showing an inverse correlation with socioeconomic status. In addition, general and abdominal obesity at 9 years are strongly associated with being classified with obesity at 4 and 6 years, so preventive interventions should be established at very early ages.

**Supplementary Information:**

The online version contains supplementary material available at 10.1186/s12887-022-03266-6.

## Background

Childhood obesity is a major public health problem in many countries [1]. Worldwide, 124 million children and 213 million children and teenagers between 5 and 19 years of age have been estimated to present obesity and overweight in 2016 [2], respectively. In Europe, the prevalence of overweight and obesity during childhood is high, with large variations between regions. A substantially higher prevalence has been observed in the Mediterranean countries, including Spain [3]. The estimated prevalence of overweight and obesity among the Spanish population aged 6 to 9 years in 2019 was 23.3% and 17.3%, respectively [4], and the prevalence of abdominal obesity in the Spanish population aged 3 to 11 years was between 21 and 26% in 2016 [5].

General obesity (GO) in childhood has been associated with higher blood pressure, myocardial dysfunction, dyslipidemia, insulin resistance, fatty liver, stigmatization, and poor school performance [6, 7]. Its tendency to persist over the years entails a greater risk of comorbidities in adulthood [8]. GO is frequently defined based on the body mass index (BMI), a parameter easy to calculate that helps diagnose overweight and obesity as well as their associations with mortality or morbidity [9, 10]. However, the use of BMI has limitations [11] since it does not allow for differentiating between lean and fat body mass and does not provide information on regional body fat distribution [12]. In children, the relationship between BMI and fat body mass varies with age and sex and must be compared with standard or reference populations [13].

Studies have shown that the waist circumference (WC), which is widely employed to define abdominal obesity (AO), is strongly associated with cardiometabolic risk factors and risk of chronic disease [14]. WC in childhood is considered an important predictor of cardiometabolic disorders, independently of BMI values [15].

In general, GO tends to appear at an early age and persists over time. Children with overweight/obesity are more likely to continue having obesity during their adolescence and adulthood. Tracking of BMI values and obesity during childhood has been well documented [16, 17]. Nevertheless, not much is known about the stability (i.e. tracking) of the AO during childhood, presumably because WC is not routinely measured in primary-care pediatric programs. Research increasingly suggests that combined measurements of BMI and WC could be a supplementary approach to detect weight fluctuations during childhood and determine the main relevant risk factors [18, 19].

The aim of this longitudinal study with a 5-year follow-up was to examine the evolution and tracking of GO and AO in the pediatric population at 4, 6, and 9 years of age and to estimate the risk of GO and AO at 9 years old relative to previous GO or AO occurrences.

## Methods

### Study design and participants

The Longitudinal Childhood Obesity Study (ELOIN for its acronym in Spanish) is a population-based cohort study in the Madrid region (6.7 million population), whose methods have been previously published [20].

The sample size was calculated to be 3,000 children of 4 years of age at the baseline, assuming a prevalence of overweight/obesity of 12% and a precision level of about ± 2%. A total of 2,901 4-year-old children were finally recruited at the baseline. Of them, 1,902 participants were included in the present analysis that underwent three physical examinations for the recording of anthropometric measurements: the 4-year-old baseline measurements during 2012–2013, the 6-year-old follow-up during 2014–2015, and the 9-year-old follow-up during 2017–2018. The information was collected in two consecutive stages: 1) standardized physical examinations performed in the health center by pediatricians and primary care nurses of the sentinel network; and 2) completion of a structured questionnaire answered by the parents through a computer-assisted telephone interview (CATI) for the collection of data on sociodemographic variables and lifestyle.

### Anthropometric measurements

The main outcome variables were abdominal obesity, measured via the WC, and general obesity, assessed with the BMI. Weight was measured with a digital scale (SECA® model 220, precision 0.1 kg), height was recorded using a telescopic stadiometer (SECA® model 220, precision 1 mm), and WC was obtained with an inextensible, locking, standardized measuring tape placed horizontally just above the iliac crest without tissue compression. All measurements were taken twice for each participant, and the mean of the two values was used for the analyses.

BMI (kg/m^2^) was adjusted by age (months) and sex according to the standardized tables of the WHO-2007 [13]. After obtaining BMI z-scores, normal weight was defined as zBMI ≤ 1 standard deviation (SD), overweight as 1 SD < zBMI ≤ 2 SD, and GO as zBMI > 2 SD [14]. WC was standardized by age (months) and sex based on reference tables of the International Diabetes Federation (IDF) for the consensus definition of metabolic syndrome. AO was defined setting the cut-off point at ≥ 90^th^ percentile [21].

Four trajectories were defined based on the persistence or variation in obesity at 4, 6, and 9 years: 1) Stable without obesity: no obesity at any of the measurements; 2) Remitting obesity at 9 years: obesity at 4 and/or 6 years, but without obesity at 9 years; 3) Incidental obesity at 9 years: obesity only at 9 years, or at 4 and 9 years, or at 6 and 9 years; and 4) Stable with obesity: obesity at all three measurements. The same trajectories were used to assess changes in both GO and AO.

### Covariates

The included sociodemographic variables, which were recorded at the baseline measurement of 4 years of age, were sex, age (months), and the household affluence estimated by the *Family Affluence Scale* (FAS) [22]. The FAS is a measure of family wealth and assets developed as an indicator of global socio-economic status. The FAS evaluates the following four items: ownership of a family car (0, 1, 2 or more), own bedroom (no = 0, yes = 1), family holidays during the past 12 months (0, 1, 2, 3 or more), and family computer(s) (0, 1, 2, 3 or more). The scores from these items are combined to produce a single index ranging from 0 (low affluence) to 9 (high affluence). The socio-economic status of respondents was classified as low (0–3 points), medium (4–5), or high (6–9), which is a categorization used in previous studies [23, 24].

### Ethics

The Ethics Committee of the University Hospital Ramón y Cajal in Madrid approved the study (CIHURC- 122/11). Prior to their participation in the current study, all participants provided written informed consent. Written informed consent was obtained from the parents or legal guardians before participation in the ELOIN study.

### Data analysis

An initial descriptive analysis was performed where the quantitative variables were summarized by their mean (SD) and the qualitative variables by percentages. Non-parametric tests were employed to determine whether the results reached statistical significance. Specifically, the Wilcoxon test was used for comparing two means while the Kruskal–Wallis test was used in cases where more than two means were compared.

The flow diagrams were represented in the form of Sankey diagrams to facilitate the visual interpretation of the tracking. The prevalence ratios (PRs) were calculated using generalized estimating equation (GEE) models under a binomial distribution. Poisson regression models were developed to estimate 1) the probability, as expressed by the relative risk (RR), of presenting GO and AO at 9 years based on the obesity status at 4 and 6 years of age, and 2) the risk of GO at 6 and 9 years relative to the presence of GO and/or AO at 4 years of age. GEE and Poisson regression models with robust variance were developed. The variables included in the models were sex, age (months), and family affluence (FAS index). The relationships of GO and AO indicators with sex and socioeconomic status were explored. Confidence intervals were established at 95% (95% CI) for all estimators. The statistical significance level was set at 0.05.

The analyses were performed using Stata v.16 (StataCorp, College Station. USA) software.

## Results

A total of 1,902 participants who completed all three measurements were included, which represented 65.5% of the participation rate over time. Of them, 49.6% were boys and the mean (SD) age was 48.5 (1.8), 73.4 (3.3), and 110.9 (4.5) months at the 4-, 6-, and 9-year-old measurements, respectively. The mean follow-up period was 5.2 years.

Weight and height were greater in boys than in girls of all ages. The values obtained for the BMI at 9 years old and for the WC at all ages were greater in the participants with low household affluence than in those with high household affluence (Table 1).

**Table 1:** Distribution of anthropometric values at 4, 6, and 9 years of age

	**N**	**Age** (months)	***p-value***	**Weight** (kg)	***p-value***	**Height** (cm)	***p-value***	**BMI** (kg/m^2^)	***p-value***	**Waist circumference** (cm)	***p-value***
		**Mean (SD)**		**Mean (SD)**		**Mean (SD)**		**Mean (SD)**		**Mean (SD)**	
**TOTAL**											
4 years	1,902	48.5 (1.8)		16.9 (2.3)		103.4 (4.4)		15.8 (1.5)		51.8 (3.8)	
6 years	1,902	73.4 (3.3)		22.4 (4.1)		117.5 (5.3)		16.1 (2.0)		56.2 (5.6)	
9 years	1,902	110.9 (4.5)		33.6 (8.0)		136.2 (6.6)		18.0 (3.3)		64.7 (8.9)	
**AGE**											
**4 years**			0.047		< 0.001		< 0.001		0.352		0.652
Boys	944	48.6 (1.9)		17.1 (2.3)		104.0 (4.4)		15.8 (1.5)		51.7 (3.6)	
Girls	958	48.4 (1.7)		16.7 (2.3)		102.9 (4.3)		15.7 (1.6)		51.9 (4.0)	
**6 years**			0.931		< 0.001		< 0.001		0.052		0.530
Boys	944	73.4 (3.3)		22.7 (4.1)		118.0 (5.4)		16.2 (2.0)		56.2 (5.3)	
Girls	958	73.4 (3.3)		22.1 (4.0)		117.1 (5.2)		16.0 (2.0)		56.2(5.8)	
**9 years**			0.901		0.006		0.001		0.086		0.098
Boys	944	110.9 (4.5)		34.1 (8.1)		136.7 (6.4)		18.1 (3.5)		65.1 (9.0)	
Girls	958	111.0 (4.5)		33.1 (7.8)		135.8 (6.7)		17.8 (3.0)		64.3 (8.7)	
**HOUSEHOLD AFFLUENCE**											
**4 years**			0.053		0.052		0.084		0.124		0.003
Low	309	48.7 (2.2)		17.3 (2.7)		103.8 (4.6)		16.0 (1.8)		52.6 (4.4)	
Medium	589	48.3 (1.6)		16.8 (2.3)		103.1 (4.1)		15.8 (1.5)		51.7 (3.8)	
High	1,004	48.5 (1.8)		16.9 (2.2)		103.5 (4.4)		15.7 (1.4)		51.6 (3.6)	
**6 years**			0.594		0.072		0.104		0.060		0.001
Low	309	73.5 (3.1)		23.0 (4.8)		117.1 (5.6)		16.5 (2.4)		57.4 (6.3)	
Medium	589	73.2 (2.9)		22.3 (4.0)		117.1 (5.1)		16.1 (2.1)		56.2 (5.8)	
High	1,004	73.4 (3.4)		22.2 (4.0)		117.7 (5.4)		16.0 (1.8)		55.9 (5.0)	
**9 years**			0.158		0.005		0.188		< 0.001		< 0.001
Low	309	110.7 (4.1)		35.2 (9.2)		136.4 (7.0)		18.7 (3.6)		66.7 (9.8)	
Medium	589	110.9 (4.1)		33.4 (8.3)		135.9 (6.5)		18.0 (3.6)		64.8 (9.3)	
High	1,004	111.1 (4.6)		33.2 (7.3)		136.4 (6.5)		17.7 (2.9)		64.0 (8.1)	

*BMI* Body mass index, *SD* Standard deviation

Table 2 shows the evolution of the standardized prevalence of overweight, GO, and AO. The prevalence of overweight between 4 and 9 years of age increased from 16.8% to 21.8% (PR: 1.30; 95% CI: 1.16–1.46), with a similar evolution by sex (PR: 1.25 in boys and 1.35 in girls) and by family affluence (PR: low affluence 1.23; medium 1.31; high 1.31). GO increased from 5.1% to 15.6% between 4 and 9 years (PR: 3.05), from 5.5% to 19.3% in boys (PR: 3.50) and from 4.7% to 11.9% in girls (PR: 2.53). A similar over-time increase in obesity was observed in the population with low, medium, and high family affluence (PR: 2.81, 3.0, and 3.26, respectively). The prevalence of GO at 6 and 9 years of age was higher in boys (11.3% and 19.3%, respectively) than in girls (6.9% and 11.9%, respectively). The prevalence was also higher in participants with low household affluence compared to those with high affluence at 4, 6, and 9 years (8.7% vs 3.9%, 14.6% vs 7.1%, and 24.6% vs. 12.6%, respectively).

**Table 2:** Evolution of the prevalence of overweight, general obesity, and abdominal obesity at 4, 6, and 9 years of age by sex and household affluence

	BMI										Waist circumference				
	Overweight^a^					General obesity^b^					Abdominal obesity^c^				
	N	%	95% CI	PR	95% CI	N	%	95% CI	PR	95% CI	N	%	95% CI	PR	95% CI
**TOTAL**															
4 years	319	16.8	(15.2–18.5)	1.00		97	5.1	(4.2–6.2)	1.00		129	6.8	(5.7–8.0)	1.00	
6 years	326	17.1	(15.5–18.9)	1.02	(0.91–1.15)	173	9.1	(7.9–10.5)	1.78	(1.51–2.11)	160	8.4	(7.2–9.7)	1,24	(1.06–1.44)
9 years	414	21.8	(20.0–23.7)	1.30	(1.16–1.46)	296	15.6	(14.0–17.3)	3.05	(2.55–3.60)	276	14.5	(13.0–16.2)	2.14	(1.82–2.51)
**SEX**															
Boys															
4 years	162	17.2	(14.9–19.7)	1.00		52	5.5	(4.2–7.2)	1.00		56	5.9	(4.6–7.6)	1.00	
6 years	175	18.5	(16.2–21.1)	1.08	(0.91–1.28)	107	11.3	(9.5–13.5)	2.06	(1.63–2.60)	70	7.4	(5.9–9.3)	1.25	(0.97–1.62)
9 years	202	21.5	(18.9–4.1)	1.25	(1.05–1.47)	182	19.3	(16.9–21.9)	3.50	(2.73–4.48)	136	14.4	(12.3–16.8)	2.43	(1.89–3.11)
Girls															
4 years	157	16.4	(14.2–18.9)	1.00		45	4.7	(3.5–6.2)	(ref)		73	7.6	(6.1–9.5)	1.00	
6 years	151	15.8	(13.6–18.2)	0.96	(0.82–1.13)	66	6.9	(5.4–8.7)	1.47	(1.16–1.86)	90	9.4	(7.7–11.4)	1.23	(1.03–1.46)
9 years	212	22.1	(19.6–24.9)	1.35	(1.14–1.59)	114	11.9	(9.8–14.1)	2.53	(1.94–3.30)	140	14.6	(12.5–17.0)	1.92	(1.55–2.37)
**HOUSEHOLD AFFLUENCE**															
Low															
4 years	51	16.5	(12.8–21.1)	1.00		27	8.7	(6.1–12.5)	1.00		36	11.6	(8.5–15.7)	1.00	
6 years	59	19.1	(15.1–23.9)	1.16	(0.86–1.55)	45	14.6	(11.0–19.0)	1.67	(1.22–2.27)	41	13.3	(10.0–17.5)	1.14	(0.86–1.50)
9 years	63	20.4	(16.2–25.3)	1.23	(0.90–1.69)	76	24.6	(20.1–29.7)	2.81	(2.02–3.90)	65	21.0	(16.8–25.9)	1.80	(1.35–2.41)
Medium															
4 years	98	16.6	(13.8–19.9)	1.00		31	5.3	(3.7–7.4)	1.00		33	5.6	(4.0–7.8)	1.00	
6 years	92	15.6	(12.9–18.8)	0.94	(0.75–1.17)	57	9.7	(7.5–12.3)	1.84	(1.38–2.45)	53	9.0	(6.9–11.6)	1.62	(1.22–2.11)
9 years	129	21.7	(18.7–25.4)	1.31	(1.05–1.62)	93	15.8	(13.0–19.0)	3.00	(2.20–4.01)	91	15.4	(12.7–18.6)	2.75	(2.01–3.78)
High															
4 years	170	16.9	(14.7–19.4)	1.00		39	3.9	(2.8–5.3)	1.00		60	6.0	(4.7–7.6)	1.00	
6 years	175	17.4	(15.2–19.9)	1.03	(0.88–1.20)	71	7.1	(5.6–8.8)	1.82	(1.38–2.39)	66	6.6	(5.2–8.3)	1.10	(0.86–1.40)
9 years	223	22.2	(19.7–24.9)	1.31	(1.12–1.54)	127	12.6	(10.7–14.8)	3.26	(2.41–4.38)	120	11.9	(10.1–14.1)	2.00	(1.57–2.55)

^a^Overweight: 1 SD < BMI ≤ 2 SD according to the WHO-2007 standardized tables^13^

^b^Obesity: BMI > 2 SD according to the WHO-2007 standardized tables^13^

^c^Abdominal obesity: ≥ 90^th^ percentile of waist circumference according to the consensus of the International Diabetes Foundation (IDF)^21^

*PR* Prevalence ratio, *95% CI* 95% confidence interval, *SD* Standard deviation

The AO prevalence increased between 4 and 9 years of age from 6.8% to 14.5% (PR: 2.14), with a fairly similar trend disaggregated by sex (PR: 2.43 in boys and 1.92 in girls) and family affluence, where the prevalence of AO was about two-fold higher in participants of low socioeconomic status versus those of high level.

Table 3 shows the variations observed in obesity at the 9-year-old follow-up. Of the included subjects, 82.4% and 82.1% were stable without GO or AO, respectively; incidental obesity was recorded in 12.1% (GO) and 11.0% (AO) of them; and 3.4% (GO) and 3.5% (AO) were stable with obesity. The prevalence of boys classified as stable without GO (78.6%) was lower than that of girls (86.1%). Participants of low socioeconomic status showed a higher prevalence of stable GO (6.2%) and AO (6.5%) than those of high status (2.3% for GO and 2.5% for AO), with children from medium-affluence households in an intermediate position.

**Table 3:** Variations in the prevalence of general and abdominal obesity in 4-, 6-, and 9-year-old children by sex and household affluence

**Obese BMI or abdominal obesity**	**General obesity**^a^			**Abdominal obesity**^b^		
	N	%	95% CI	N	%	95% CI
**TOTAL**						
Stable without obesity^c^	1,567	82.4	(80.6–84.0)	1,562	82.1	(80.3–83.8)
Remitting obesity at 9 years^d^	39	2.1	(1.5–2.8)	64	3.4	(2.6–4.3)
Incidental obesity at 9 and (4 or 6)^e^	230	12.1	(10.7–13.6)	209	11.0	(9.6–12.5)
Stable with obesity^f^	66	3.4	(2.7–4.4)	67	3.5	(2.8–4.5)
**SEX**						
**Boys**						
Stable without obesity^c^	742	78.6	(75.9–81.1)	780	82.6	(80.1–84.9)
Remitting obesity at 9 years^d^	20	2.1	(1.4–3.3)	28	3.0	(2.1–4.3)
Incidental obesity at 9 and (4 or 6)^e^	145	15.4	(13.2–17.8)	112	11.9	(10.0–14.1)
Stable with obesity^f^	37	3.9	(2.9–5.4)	24	2.5	(1.7–3.8)
**Girls**						
Stable without obesity^c^	825	86.1	(83.8–88.2)	782	81.6	(79.0–84.0)
Remitting obesity at 9 years^d^	19	2.0	(1.3–3.1)	36	3.8	(2.7–5.1)
Incidental obesity at 9 and (4 or 6)^e^	85	8.9	(7.2–10.8)	97	10.1	(8.3–12.2)
Stable with obesity^f^	29	3.0	(2.1–4.3)	43	4.5	(3.3–6.0)
**HOUSEHOLD AFFLUENCE**						
**Low**						
Stable without obesity^c^	226	73.1	(67.9–77.8)	229	73.0	(68.9–78.7)
Remitting obesity at 9 years^d^	7	2.3	(1.1–4.7)	15	5.4	(2.9–7.9)
Incidental obesity at 9 and (4 or 6)^e^	57	18.5	(14.5–23.2)	45	15.1	(11.0–19.0)
Stable with obesity^f^	19	6.2	(3.9–9.5)	20	6.5	(4.2–9.8)
**Medium**						
Stable without obesity^c^	487	82.7	(79.4–85.5)	484	82.1	(78.9–85.1)
Remitting obesity at 9 years^d^	9	1.5	(0.8–2.9)	14	2.5	(1.4–4.0)
Incidental obesity at 9 and (4 or 6)^e^	69	11.7	(9.4–14.6)	70	12.0	(9.5–14.8)
Stable with obesity^f^	24	4.1	(2.7–6.0)	21	3.4	(2.3–5.4)
**High**						
Stable without obesity^c^	854	85.1	(82.7–87.1)	849	84.8	(82.2–86.7)
Remitting obesity at 9 years^d^	23	2.3	(1.5–3.4)	35	3.4	(2.5–4.8)
Incidental obesity at 9 and (4 or 6)^e^	104	10.4	(8.6–12.4)	94	9.3	(7.7–11.3)
Stable with obesity^f^	23	2.3	(1.5–3.4)	26	2.5	(1.8–3.8)

^a^General obesity: BMI > 2 SD according to the WHO-2007 standardized tables^13^

^b^Abdominal obesity: ≥ 90^th^ percentile of waist circumference according to the consensus of the International Diabetes Federation (IDF)^21^

^c^No obesity at any of the measurements

^d^Obesity at 4 and/or 6 years but not at 9 years

^e^Obesity only at 9 years, or at 4 and 9 years, or at 6 and 9 years

^f^Obesity at all three measurements

*95% CI* 95% Confidence interval

**Fig. 1:**
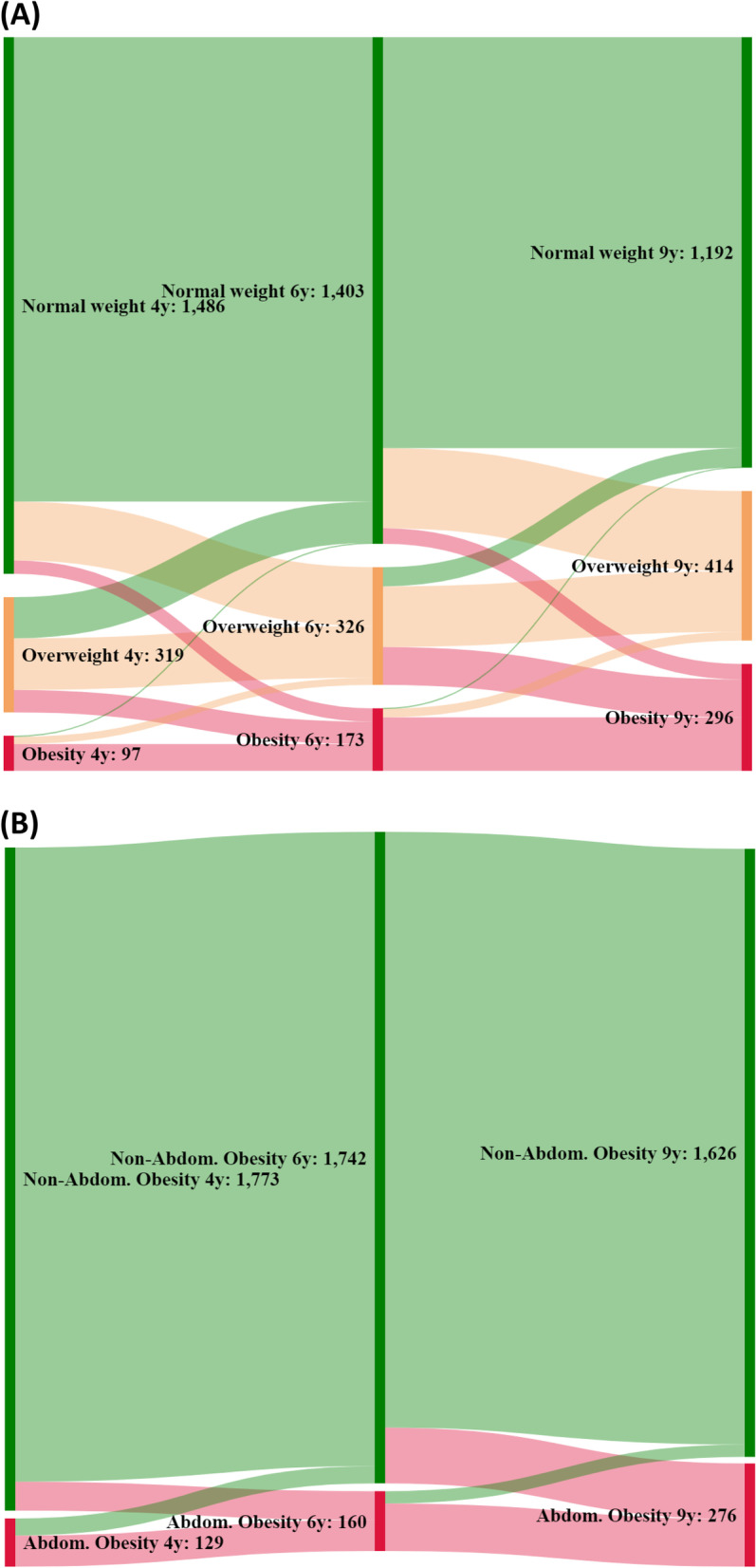
Sankey diagram. Weight status transitions at 4, 6, and 9 years of age. Classification based on body mass index: normal weight, overweight, and obesity according to WHO-2007 criteria^13^ (**A**); abdominal obesity (**B**) according to International Diabetes Federation consensus criteria^21^

The Sankey diagram (Fig. 1) displays the tracking of obesity throughout the three measurements (see also Figs. 1S and 2S in Supplementary material). Most of the children with GO or AO at 4 or 6 years old remained in the same condition at 9 years old; 77.3% and 63.6% of children with GO and AO at 4 years of age also showed GO or AO at 9 years, respectively. Only 3.1% and 0.6% of those who were classified with GO at 4 and 6 years, respectively, remitted to normal weight at 9 years. Of those classified with obesity at 9 years, nearly half were determined to be incidental cases.

Table 4 shows the RRs of GO and AO at 9 years old relative to participants stable without obesity. The RR of GO was 4.61 (95% CI: 2.76–7.72) for participants who presented obesity at the age of 4 but not at the age of 6, 9.36 (95% CI: 7.72–11.35) if they were classified with obesity at 6 years but not at 4, and 10.27 (95% CI: 8.52–12.37) if they presented obesity at both 4 and 6 years of age. Similar results were found for the risk of presenting AO at 9 years, with estimated RRs of 4.14 (95% CI: 2.65–6.48), 9.56 (95% CI: 7.79–11.74), and 9.88 (95% CI: 8.07–12.11) for participants with obesity at the age of 4 but not at 6, with obesity at 6 years but not at 4, or with obesity at both 4 and 6 years of age, respectively.

**Table 4:** Risk of general and abdominal obesity at 9 years of age relative to the presence of obesity at 4 and 6 years of age

	General obesity^a^ at 9 years				Abdominal obesity^b^ at 9 years			
	N	RR^c^	95% CI	*p*-value	N	RR^c^	95% CI	*p*-value
**Obesity at 4 and/or 6 years**								
Stable without obesity at 4 and 6 years	1,706	1.00			1,696	1.00		
Obesity at 4 but not at 6 years	23	4.61	(2.76–7.72)	< 0,001	46	4.14	(2.65–6.48)	< 0,001
No obesity at 4 but obesity at 6 years	99	9.36	(7.72–11.35)	< 0,001	77	9.56	(7.79–11.74)	< 0,001
Obesity at both 4 and 6 years	74	10.27	(8.52–12.37)	< 0,001	83	9.88	(8.07–12.11)	< 0,001

^a^General obesity: BMI > 2 SD according to the WHO-2007 standardized tables^13^

^b^Abdominal obesity: ≥ 90^th^ percentile of waist circumference according to the consensus of the International Diabetes Federation (IDF)^21^

^c^Relative risk estimated by Poisson regression and adjusted by age, sex, and household affluence

*95% CI* 95% Confidence interval

Finally, participants with combined obesity of GO and AO at 4 years old showed RRs of GO of 16.10 (95% CI: 12.41–20.87) at 6 years and 6.54 (95% CI: 5.40–7.91) at 9 years, relative to children without obesity at 4 years old. Children with only GO at 4 years old had RRs of a BMI representative of obesity of 12.37 (95% CI: 8.92–17.17) and 5.65 (95% CI: 4.29–7.43) at the ages of 6 and 9, respectively, relative to those who had AO but not GO (Table 5).

**Table 5:** Risk of general obesity at 6 and 9 years of age relative to the presence of general obesity and/or abdominal obesity at 4 years of age

	General obesity^a^ at 6 years				General obesity^a^ at 9 years		
	N	RR^c^	95% CI	*p*-value	RR^c^	95% CI	*p*-value
**Obesity at 4 years**							
No general obesity and no abdominal obesity	1,741	1.00			1.00		
General obesity but no abdominal obesity	32	12.37	(8.92–17.17)	< 0,001	5.65	(4.29–7.43)	< 0,001
Abdominal obesity but no general obesity	64	5.19	(3.19–8.44)	< 0,001	3.15	(2.17–4.57)	< 0,001
Both general obesity and abdominal obesity^b^	65	16.10	(12.41–20.87)	< 0,001	6.54	(5.40–7.91)	< 0,001

^a^General obesity: BMI > 2 SD according to the WHO-2007 standardized tables^13^

^b^Abdominal obesity: ≥ 90^th^ percentile of waist circumference according to the consensus of the International Diabetes Federation (IDF)^21^

^c^Relative risk estimated by Poisson regression and adjusted by age, sex, and household affluence

*95% CI* 95% Confidence interval

## Discussion

This study describes the longitudinal changes in the GO and AO condition in children from 4 to 9 years of age. A high increase in GO and AO over time was evident from the large number of incidental cases at 6 and 9 years old and low remission percentage, with prevalence rates three-fold (GO) and two-fold higher (AO) at 9 years old than those observed at 4 years old and with similar tendencies across all socio-economic levels. Additionally, the children who were already classified with obesity at 4 or 6 years old presented a higher risk of maintaining their obesity condition at 9 years old. These results highlight the importance of the early detection of changes in obesity (general and abdominal) since its appearance at 4 or 6 years of age is highly determinant of obesity at 9 years.

This study estimated a prevalence of GO of 5.1%, 9.1%, and 15.6% at 4, 6, and 9 years, respectively. The present results were of a similar magnitude and trend to other cross-sectional studies in our setting. A greater prevalence of GO has also been observed in boys than in girls [4]. Our results are consistent with other studies that reported that childhood is a particularly susceptible period for the development of obesity, especially after 5 years of age [25, 26].

The prevalence of AO increased with age and was similar to that observed by Schröeder et al., which reported a prevalence of abdominal obesity of 13% among Spanish children aged 6 to 11 years [27], but lower than the estimated prevalence of 21.5% at 3 to 8 years recently reported by Aranceta-Bartrina et al. [5]. The different measurement methods and cut-off points for the WC used to define AO could explain these differences.

A clear correlation between socioeconomic status and childhood obesity is observed in Western countries, with higher prevalence associated with lower socioeconomic levels [28]. However, whether these differences remain stable or continue to increase is not clear, with some studies describing an increasing inequality while others note a fairly uniform evolution [29]. The present study found higher prevalence rates in children with low household affluence, with similar trends for both GO and AO over the ages of 4 to 9 years in low-, medium-, and high-affluence families. Highly disparate trends have been observed in Spain. The study by Albaladejo-Vicente et al. [30] examined the population aged 5–15 years based on the national health survey from 1997 to 2017 and showed a decreasing trend in obesity in children of parents of low socioeconomic status and an increasing trend in girls. A study in Andalusia in 2011–2012 observed a gradient of childhood obesity by socioeconomic indicators that disappeared by 2015–2016 [31]. In contrast, an ecological study conducted in Catalonia based on primary care electronic medical records noted an increase in inequality between 2006 and 2016 [32].

On the other hand, our study tracked GO from 4 to 6 to 9 years of age and the findings were consistent with several studies that also suggest the stability and persistence of GO throughout childhood [33, 34]. Children with or without GO at 4 or 6 years old tended to remain in the same category at 9 years. Furthermore, over 60% of participants who had GO at 4 or 6 years continued to have obesity at 9 years. As shown in the Sankey diagram and Figure S1, only 3% and 0.6% of the children who presented GO at 4 or 6 years of age, respectively, remitted to normal weight at 9 years.

The risk of GO at 9 years was also greater if they presented obesity at both 4 and 6 years of age than if they did so at only one of the previous measurement points. Other studies reported similar results. A study in Norway found that children with excess weight at 2–4 years of age had an 11-fold higher risk (odds ratio) of being overweight at 5–7 years than children with normal weight [35]. In a study in Australia, 5-year-old children who were classified with obesity were 25 times more likely to continue having obesity 3 years later than those who were not [36].

The information available for the assessed age group is insufficient to compare the variations and risk of AO, not only in Spain but also in other countries. Vogelezang et al. [37] found that the persistence of AO was very high as early as at 2 and 6 years of age. A recent study by Ochiai et al. [38] estimated that half of the children classified with AO remained in the same category during adolescence, and Chrzanowska et al. reported that 51% of children with AO at the age of 7 remained with AO at 15 years [39]. The present study observed an even greater persistence of AO: 60–80% of children who presented obesity at 4 or 6 years old remained in the same category at 9 years (see supplementary material) and the risk of AO at 9 years was ten times higher if they had obesity at both 4 and 6 years of age. These results are in agreement with other studies, but comparisons should be made with caution given the differences in the follow-up periods, ages, and classification methods used [40, 41].

An aspect of important public health implications is the greater probability of finding GO in children of 6 or 9 years of age who presented AO but not GO at the age of 4, which represented 3.4% of the analyzed sample. Considering the high cardiometabolic risk in children with normal weight and BMI but with AO, the combined measures of weight, height, and WC will improve the predictive power to detect this health problem [42].

To correctly interpret the results of this study, several limitations must be taken into consideration. On the one hand, no information was available on the weight status between birth and 4 years, which prevents the completion of the children’s obesity trajectories. In the constitution of the ELOIN cohort, a moderate bias was observed in the selection of the sample that could affect its population representativeness: children with low educational level and foreign parents had a lower response rate at the baseline [20]. However, the sociodemographic and anthropometric variables were quite similar between those who attended the three follow-up measurements and the characteristics of the cohort at the baseline (Table 1S in Supplementary material).

The longitudinal design must be highlighted among the strengths of the study. In addition, the sample is representative of the population of the Community of Madrid, even with the above-mentioned selection bias. The measurements of anthropometric variables were based on objective criteria and performed in a standardized way, hence minimizing validity errors, such as self-reported measures or those provided by parents [43].

## Conclusions

The present study showed that both GO and AO begin at an early age, are associated with low socioeconomic status, and increase rapidly with age. The follow-up of the participants from 4 to 9 years of age revealed that, once instituted, GO and AO tend to persist strongly throughout early to middle childhood. Both forms of obesity at the age of 9 years are closely related to previous obesity conditions at 4 or 6 years of age. Therefore, prevention and management interventions should be established at very early ages. GO and AO are complementary indicators and their combined use could maximize the detection and control of this health problem.

## Supplementary Information

**Additional file 1:** **Figure 1S**. Weight status transitions (normal weight, overweight, and obesity) classified according to WHO-2007 criteria13: (A) from 4 to 9 years of age, and (B) from 6 to 9 years of age. **Figure 2S**. Abdominal obesity transitions based on waist circumference following the International Diabetes Federation consensus criteria21: (A) from 4 to 9 years of age, and (B) from 6 to 9 years of age.

**Additional file 2:** **Table 1S**. Characteristics of children enrolled in the original cohort and in the study sample.

## Data Availability

According to private and confidential clauses stated in the informed consent, the dataset generated and analyzed during the current study is restricted and not publicly available for ethical reasons. Data will be available from Dr. Honorato Ortiz-Marrón (E-mail: honorato.ortiz@salud.madrid.org upon reasonable request.

## Abbreviations

BMI: Body mass index
CI: Confidence interval
PR: Prevalence ratio
RR: Relative risk
SD: Standard deviation
WC: Waist circumference
AO: Abdominal obesity
GO: General obesity
WHO: World Health Organization
IDF: International Diabetes Foundation
ELOIN: Longitudinal Childhood Obesity Study
