# Supplementary material for: Tracking and risk of abdominal and general obesity in children between 4 and 9 years of age. The Longitudinal Childhood Obesity Study (ELOIN)
Source: BMC Pediatr. 2022 Apr 12;22:198. doi: 10.1186/s12887-022-03266-6 (PMC9004048; doi:10.1186/s12887-022-03266-6)
Supplement: Supplementary file 1 — Additional file 1: Figure 1S. Weight status transitions (normal weight, overweight, and obesity) classified according to WHO-2007 criteria13: (A) from 4 to 9 years of age, and (B) from 6 to 9 years of age. Figure 2S. Abdominal obesity transitions based on waist circumference following the International Diabetes Federation consensus criteria21: (A) from 4 to 9 years of age, and (B) from 6 to 9 years of age. [file 12887_2022_3266_MOESM1_ESM.docx]

(A)

(B)

**Figure 1S. Weight status transitions (normal weight, overweight, and obesity) classified according to WHO-2007 criteria^13^: (A) from 4 to 9 years of age, and (B) from 6 to 9 years of age.**

(A)

(B)

**Figure 2S. Abdominal obesity transitions based on waist circumference following the International Diabetes Federation consensus criteria^21^: (A) from 4 to 9 years of age, and (B) from 6 to 9 years of age.**
