# Supplementary material for: Tracking and risk of abdominal and general obesity in children between 4 and 9 years of age. The Longitudinal Childhood Obesity Study (ELOIN)
Source: BMC Pediatr. 2022 Apr 12;22:198. doi: 10.1186/s12887-022-03266-6 (PMC9004048; doi:10.1186/s12887-022-03266-6)
Supplement: Supplementary file 2 — Additional file 2: Table 1S. Characteristics of children enrolled in the original cohort and in the study sample. [file 12887_2022_3266_MOESM2_ESM.docx]

**Table 1S. Characteristics of children enrolled in the original cohort and in the study sample.**

|  | **Original cohort** | | | **Study sample** | | | |
| --- | --- | --- | --- | --- | --- | --- | --- |
|  | **n** | **Mean (SD) / %** | **95% CI** | **n** | **Mean (SD) / %** | **95% CI** |  |
| **Age (months)** | 2,901 | 48.5 (1.8) | (48.5–48.6) | 1,902 | 48.4 (1.8) | (48.4–48.6) |  |
| **Sex** |  |  |  |  |  |  |  |
| Boys | 1,465 | 50.5 | (48.7–52.6) | 944 | 49.6 | (47.4–51.9) |  |
| Girls | 1,436 | 49.5 | (47.7–51.3) | 958 | 50.4 | (48.1–52.6) |  |
| **Household affluence*** |  |  |  |  |  |  |  |
| Low | 571 | 19.7 | (18.2–21.2) | 309 | 16.2 | (14.6–18.0) |  |
| Medium | 876 | 30.2 | (28.5–31.9) | 589 | 31.0 | (28.9–33.1) |  |
| High | 1,452 | 50.1 | (48.2–51.9) | 1,004 | 52.8 | (50.5–55.1) |  |
| **General obesity*** |  |  |  |  |  |  |  |
| Yes | 2,720 | 93.9 | (93.0–94.7) | 1,805 | 94.9 | (93.8–95.9) |  |
| No | 177 | 6.1 | (5.3–7.0) | 97 | 5.1 | (4.2–6.2) |  |
| **Abdominal obesity** |  |  |  |  |  |  |  |
| Yes | 2,668 | 92.0 | (90.9–92.9) | 1,773 | 93.2 | (92.0–94.3) |  |
| No | 233 | 8.0 | (7.1–9.1) | 129 | 6.8 | (5.7–8.0) |  |
| SD: standard deviation.  * Variables with missing values. | | | | | | | |
